# Comment on intranasal esketamine combined with oral midazolam provides adequate sedation for outpatient pediatric dental procedures

**DOI:** 10.1097/JS9.0000000000001739

**Published:** 2024-05-29

**Authors:** Kejia Chu, Haihui Wang, Zhijun Zheng, Juxiang Peng, Juan Gao

**Affiliations:** a Department Orthodontics, Guiyang Hospital of Stomatology; b Department Pediatric Dentistry, Guiyang Hospital of Stomatology, Guiyang, People’s Republic of China

*Dear Editor,*

We read an article entitled ‘Intranasal esketamine combined with oral midazolam provides adequate sedation for outpatient pediatric dental procedures: a prospective cohort study’ published in the *International Journal of Surgery* ^1^. This prospective cohort study explores the sedation effects of intranasal esketamine combined with oral midazolam during outpatient procedures in children. The study was conducted well, and the results suggest that using midazolam oral solution combined with esketamine nasal drops is a considerable method for noninvasive sedation following a preoperative anxiety scale evaluation for children aged 2–6 years with dental anxiety who require dental surgery. However, there were some issues that need to be addressed.

First, we found an error in the age data presented in Table 1; the mean ages should be 5.1, 5.0, and 5.7 instead of 51.5, 50, and 57, respectively. There is also a mistake in the median (IQR) of age.

Second, the sample size of just 60 is very small for a cohort study, and no sample size estimation was provided in the trial report to demonstrate that there was enough sample size to obtain credible results^2^.

Third, the data on dental procedures in the table of characteristics of participants and the table of security indicators was missing. It is important to know the types of dental diseases and their relative procedures in children, as these data are critical to the failure or success of the anesthesia intervention.

## Ethical approval

Our submitted manuscript does not involve any patients without the ethical approval document.

## Consent

Our submitted manuscript does not involve any patients without the written informed consent documents.

## Sources of funding

This study was supported by the Guiyang Science and Technology Plan Project (2022-4-12-6) and the Science and Technology Fund of Guizhou Provincial Health and Family Planning Commission (gzwjkj2014-1-021).

## Author contribution

K.C., J.P., and Z.Z.: participated in the original idea and writing the first draft; H.W. and J.G.: edited the final manuscript. All authors approved the last version of this manuscript.

## Conflicts of interest disclosure

There are no conflicts of interest among the authors.

## Research registration unique identifying number (UIN)

Not applicable.

## Guarantor

Haihui Wang.

## Data availability statement

The supporting data used in the paper can be obtained via the requirement from the authors.

## Provenance and peer review

Commentary, internally reviewed.

## References

[1] Wang J Zeng J Zhao N . Intranasal esketamine combined with oral midazolam provides adequate sedation for outpatient pediatric dental procedures: a prospective cohort study. Int J Surg 2023;109:1893–1899. 37288546 10.1097/JS9.0000000000000340 PMC10389564

[2] Pizzi C De Stavola B Merletti F . Sample selection and validity of exposure-disease association estimates in cohort studies. J Epidemiol Community Health 2011;65:407–11. 20881022 10.1136/jech.2009.107185

